# Temporal Changes in Gene Expression Profile during Mature Adipocyte Dedifferentiation

**DOI:** 10.1155/2017/5149362

**Published:** 2017-03-19

**Authors:** Julie Anne Côté, Frédéric Guénard, Julie Lessard, Marc Lapointe, Simon Biron, Marie-Claude Vohl, André Tchernof

**Affiliations:** ^1^Centre de Recherche de l'Institut Universitaire de Cardiologie et de Pneumologie de Québec, 2725 Chemin Ste-Foy, Quebec City, QC, Canada G1V 4G5; ^2^Centre de Recherche du CHU de Québec, 2705 Boulevard Laurier, Quebec City, QC, Canada G1V 4G2; ^3^École de Nutrition, Université Laval, 2425 Rue de l'Agriculture, Quebec City, QC, Canada G1V 0A6; ^4^Institut sur la Nutrition et les Aliments Fonctionnels (INAF), 2440 Boulevard Hochelaga, Quebec City, QC, Canada G1V 0A6

## Abstract

*Objective*. To characterize changes in gene expression profile during human mature adipocyte dedifferentiation in ceiling culture.* Methods*. Subcutaneous (SC) and omental (OM) adipose tissue samples were obtained from 4 participants paired for age and BMI. Isolated adipocytes were dedifferentiated in ceiling culture. Gene expression analysis at days 0, 4, 7, and 12 of the cultures was performed using Affymetrix Human Gene 2.0 STvi arrays. Hierarchical clustering according to similarity of expression changes was used to identify overrepresented functions.* Results*. Four clusters gathered genes with similar expression between day 4 to day 7 but decreasing expression from day 7 to day 12. Most of these genes coded for proteins involved in adipocyte functions (*LIPE*,* PLIN1*,* DGAT2*,* PNPLA2*,* ADIPOQ*,* CEBPA*,* LPL*,* FABP4*,* SCD*,* INSR*, and* LEP*). Expression of several genes coding for proteins implicated in cellular proliferation and growth or cell cycle increased significantly from day 7 to day 12 (*WNT5A*,* KITLG*, and* FGF5*). Genes coding for extracellular matrix proteins were differentially expressed between days 0, 4, 7, and 12 (*COL1A1*,* COL1A2*, and* COL6A3*,* MMP1*, and* TGFB1*).* Conclusion*. Dedifferentiation is associated with downregulation of transcripts encoding proteins involved in mature adipocyte functions and upregulation of genes involved in matrix remodeling, cellular development, and cell cycle.

## 1. Introduction

The process of preadipocyte differentiation was viewed as an irreversible and terminal event leading to the formation of mature adipocytes. However, in vitro studies have shown that mature adipocytes are able to return to a more primitive phenotype when they are subjected to ceiling culture [1]. In previous studies, we have shown that the dedifferentiation process is relatively independent of the fat depot, obesity level, sex, or age of the cell donor [2]. The fibroblast-like cells resulting from the process have a great potential for proliferation [1]. Dedifferentiated fat (DFAT) cells express embryonic stem cell markers in addition to being multipotent. Indeed, when cultured under appropriate conditions these cells may redifferentiate into lipid-storing adipocytes, osteocytes, chondrocytes, or even other cell types outside of the mesenchymal lineage [1, 3–5]. Thus, ceiling culture represents an interesting model to study adipocyte biology and its role in metabolic homeostasis, especially the process of adipocyte dedifferentiation [6].

**Figure 1:**
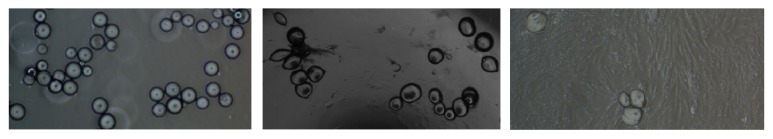
Phase contrast microscopy of human subcutaneous mature adipocytes undergoing dedifferentiation at various stages (4x) in ceiling culture at days 4, 7, and 12.

In past studies, we observed important morphological changes during the dedifferentiation process. Cells lose their round shape and become elongated as they shed their lipid content. At day 12 of the process, cells have a fibroblastic appearance (Figure 1) [2]. In addition to morphological changes, some authors observed important modifications in the expression of genes associated with the adipogenic and lipogenic programs of mature adipocytes in DFAT cells. The expression of lipoprotein lipase* (LPL)*, leptin* (LEP)*, glucose transporter type 4* (GLUT4)*, peroxisome proliferator-activated receptor gamma* (PPARγ)*, and CCAAT/enhancer binding protein alpha* (CEBPA)* was significantly decreased or even undetected in DFAT cells compared to mature adipocytes [5]. We have previously corroborated these results by showing a significant decrease in* PPARγ*,* CEBPA*, and adiponectin* (ADIPOQ)* gene expression in DFAT cells compared to mature adipocytes. We also demonstrated that expression of genes encoding proteins implicated in extracellular matrix (ECM) remodeling including matrix-metalloproteinase 1* (MMP1)*, fibroblast activated-protein* (FAP)*, dipeptidyl peptidase IV* (DPP4)*, and transforming growth factor *β*1* (TGFβ1)* was significantly upregulated during the dedifferentiation process [2].

Even if the process of dedifferentiation and the resulting DFAT cells have been characterized over the past decade, little is known about the in vitro inducer and the temporal modifications underlying this process. Furthermore, some have wondered whether dedifferentiation does occur in vivo. In 2013, Tata et al. demonstrated that differentiated airway epithelial cells were able to revert into stable and functional stem cells in vivo [7]. In 2015, results from a study by Cinti et al. using markers of endocrine lineage, *β*-cell-specific transcription factor, and a newly identified endocrine progenitor cell marker showed that *β*-cells dedifferentiated and converted to *α*- and *δ*-like cells in human type 2 diabetes [8]. Still, there is no evidence of adipocyte dedifferentiation in vivo and more studies are needed to understand the process. Most studies on dedifferentiation have examined differences in gene expression between mature adipocytes and cells once they were dedifferentiated. We aimed to understand the physiological process of human mature adipocyte dedifferentiation by characterizing temporal changes in gene expression during ceiling culture. More specifically, the objective was to identify genes or groups of genes that are modulated during the process using Affymetrix gene expression microarrays. We hypothesized that mature adipocytes gradually lose their mature adipocyte signature during dedifferentiation and that these changes are associated with an upregulation of genes associated with the cell cycle and the ECM.

## 2. Methods

### 2.1. Tissue Sampling

Adipose tissue samples were obtained from 2 men and 2 women undergoing bariatric surgery as a treatment for severe obesity. They were paired for age and BMI (mean age: 51 years; mean BMI: 48 kg/m^2^). Blood lipid values of the patients and medication used are shown in Supplemental Table  1 in Supplementary Material available online at https://doi.org/10.1155/2017/5149362. Samples were collected at the time of the surgery from two different abdominal fat depots: the greater omentum (OM) and the abdominal subcutaneous (SC) fat compartment. Consent was obtained through the management framework of the* Institut Universitaire de Cardiologie et de Pneumologie de Québec* Obesity Tissue Bank. Portions of adipose tissues were quickly frozen in liquid nitrogen and fixed in 10% formalin for paraffin embedding. The remainder of the tissue was digested by collagenase as previously described by our group. Briefly, tissue was digested with collagenase type I in Krebs-Ringer-Henseleit (KRH) buffer for up to 45 minutes at 37°C according to a modified version of the Rodbell method [9]. Adipocyte suspensions were filtered through nylon mesh and washed 3 times with KRH buffer. The residual KRH buffer of adipocyte isolation, which contained the stromal-vascular fraction, was centrifuged and the pellet was washed in DMEM-F12 culture medium supplemented with 10% calf serum, 2.5 *μ*g/mL amphotericin B, and 50 *μ*g/mL gentamicin. Isolated mature adipocytes were used for ceiling culture.

### 2.2. Ceiling Culture

Isolated mature adipocytes were counted and 500,000 cells were added to a T-25 flask completely filled with DMEM-F12 supplemented with 20% calf serum. Flasks were incubated upside down at 37°C, 5% CO_2_. For gene expression analysis, OM and SC ceiling culture from each patient were harvested at day 4 and day 7. One flask per depot per patient was reversed at day 7 and maintained for an additional 5 days in standard culture until day 12 in the same medium. Time points were chosen based on our observations that harvesting cells at day 4 provides a round cell population that has completely adhered to the flask while day 7 corresponds to the time point where the flasks are reversed. At day 12, the majority of cells are fibroblast-like cells.

### 2.3. Gene Array Analyses

For each time point, total RNA was isolated from cultured OM and SC cells. Quantity of total RNA was measured using a NanoDrop ND-1000 Spectrophotometer (NanoDrop Technologies, Wilmington, DE, USA). Total RNA quality was assayed on an Agilent BioAnalyzer (Agilent Technologies, Santa Clara, CA, USA). DNA microarray analyses were carried out with Affymetrix Human Gene 2.0 ST according to the Affymetrix standard protocol. The Affymetrix Human Gene 2.0 ST v1 array interrogates >36,000 transcripts and targets >21,000 RefSeq genes. Total RNA (100 ng per sample) was labeled using the Affymetrix GeneChip® WT cDNA Synthesis and Amplification Kit. ARN was then hybridized to the arrays following the manufacturer instructions (Affymetrix, Santa Clara, CA). The cRNA hybridization cocktail was incubated overnight in a rotating hybridization oven at 45°C. After 16 h of hybridization, we removed the cocktail and the arrays were washed. They were stained in an Affymetrix GeneChip fluidics station 450 according to the Affymetrix protocol and scanned using the Affymetrix GCS 3000 7G and the GeneChip Command Console Software (AGCC) (Affymetrix, Santa Clara, CA) to produce the probe cell intensity data (CEL). The Affymetrix Expression Console Software was used to analyze image data and quality control was performed. The method of Robust Multiarray Analysis (RMA) was used to perform background subtraction and to normalize probe set intensities [10]. Differentially expressed transcripts were identified by analysis of variance (ANOVA) across time points using the Transcriptome Analysis Console v2.0. Pairwise comparisons between dedifferentiation days 0 and 4 and days 4 and 7 as well as between days 7 and 12 were further tested to identify significant changes in expression levels (false-discovery rate- [FDR-] corrected *P* values ≤ 0.05). Changes in expression levels were expressed as fold changes.

### 2.4. Principal Component Analysis and Clustering

Principal component analysis was conducted to represent sample distributions through the dedifferentiation process using R prcomp and pca3d packages (R Development Core Team: R: A language and environment for statistical computing. Vienna, Austria: R Foundation for Statistical Computing; 2008. http://www.R-project.org) from normalized expression data of 33,297 transcripts accurately measured in all samples. Hierarchical clustering was used to group differentially expressed transcripts with annotated genes in clusters according to similarity of changes (FDR-corrected significant changes) in expression levels between dedifferentiation days 4 and 7 as well as between days 7 and 12. Clustering was conducted with the Cluster 3.0 [11] software using Euclidean distance and average linkage clustering and further visualized using Treeview 3.0 [12].

### 2.5. Gene Function Enrichment Analyses

Lists of genes from each cluster composed of more than 10 genes were submitted to the Ingenuity Pathway Analysis (IPA) system for gene function enrichment analysis. Genes from each cluster were classified according to functions and *P* values for overrepresentation were calculated using a right-tailed Fisher's exact test. Functions overrepresented among each cluster were then identified.

### 2.6. Messenger RNA Expression by Quantitative Real-Time PCR

Quantitative real-time PCR measurements were performed by the CHU de Québec Research Center Gene Expression Platform (Québec, QC, Canada). First, complementary DNA was generated from total RNA using random hexamers, oligo dT18, and Superscript III RNase H-RT (Invitrogen Life Technologies, Burlington, ON, Canada). It was purified using the QIAquick PCR Purification Kit (Qiagen, Hilden, DE). The LightCycler 480 (Roche Diagnostics, Indianapolis, IN, USA) and the SYBRGreen I Master (Roche Diagnostics, Indianapolis, IN, USA) were used to perform real-time cDNA amplification in duplicate. PCR reactions were as follows: 45 cycles, denaturation at 95°C for 10 sec, annealing at 60°C for 10 sec, elongation at 72°C for 14 sec, and then reading at 74°C for 5 sec. To assess nonspecific signal, a melting curve was performed. The number of copies for each transcript was calculated according to Luu-The et al. [13] using the second derivative method and a standard curve of* Cp* versus logarithm of the quantity. A standard curve was established using known amounts of purified PCR products and the LightCycler 480 v1.5 program provided by the manufacturer (Roche Diagnostics, Mannheim, DE) [14]. The efficiency of PCR amplification was verified. Target gene amplifications were normalized using 3 validated housekeeping genes, ATP synthase O subunit (ATP5O), Glucuronidase Beta (GUSB), and Heat Shock Protein 90 Alpha Family Class B Member (HSP90AB1) [15]. The 3 genes exhibited stable expression levels so only results with ATP5O are shown. Primer sequences were designed using Gene Tools 2.0 software (Biotools Inc., Edmonton, AB, Canada) and their specificity was verified by blast in the GenBank database. The synthesis was performed by IDT (Integrated DNA Technology, Coralville, IA, USA). The following sequences were used for quantitative PCR (forward/reverse):* ATP5O*: 5′-AACGACTCCTTGGGTATTGCTTAA-3′/5′ATTGAAGGTCGCTATGCCACAG-3′,* ADIPOQ*: 5′-TTTGGAGTGTTGGTAGGTGTCTGT-3′/5′GGGACATAGGTAAGAGGAAGTAGAGT-3′,* LIPE*: 5′-GAAGACTCTGCAGGGATCCAATA-3′/5′TTTGGATGTAAGGTGATTGCTGTGG-3′, Wingless-type family member 5A* (WNT5A)*: 5′-CCAGTTCAAGACCGTGCAGAC-3′/5′CACACAAACTGGTCCACGATCTC-3′, Fibroblast growth factor 5 (FGF5): 5′-CTTGAACAGCTGGAGGAATACATTTTA3′/5′TGGGACTTGGCATGGATAACTGTT-3′,* MMP1*: 5′-ACATGCGCACAAATCCCTTCTAC3′/5′CTTGGGGTATCCGTGTAGCA-3′,* MMP2*: 5′-GGGACAAGAACCAGATCACATACAG-3′/5′CGAGCAAAGGCATCATCCACT-3′,* MMP3*: 5′-CTGCTTTGTCCTTTGATGCTGTC3′/5′AAACGAGGTCCTTGCTAGTAACTTCA-3′.

### 2.7. Statistical Analyses

Statistical analyses were performed using JMP 12 software. For hierarchical clustering, changes in expression levels of genes between dedifferentiation time points were measured as fold changes between gene expression on day 4 versus day 7 as well as gene expression between day 7 and day 12. For gene expression measurements, graph bars represent mean values of percentage of mRNA relative expression versus housekeeping (HK) gene and error bars are the standard error of the mean (SEM). Changes in expression levels between day 7 and day 12 were assessed using the Wilcoxon matched-paired.

## 3. Results

### 3.1. Morphological Changes

The cellular morphology of dedifferentiating adipocytes is illustrated in Figure 1. On day 4, the cells are generally round and adherent while on day 7, they are elongated and show multiple lipid droplets. On day 12, most of the cells display a fibroblast-like morphology.

### 3.2. Principal Component Analysis

**Figure 2:**
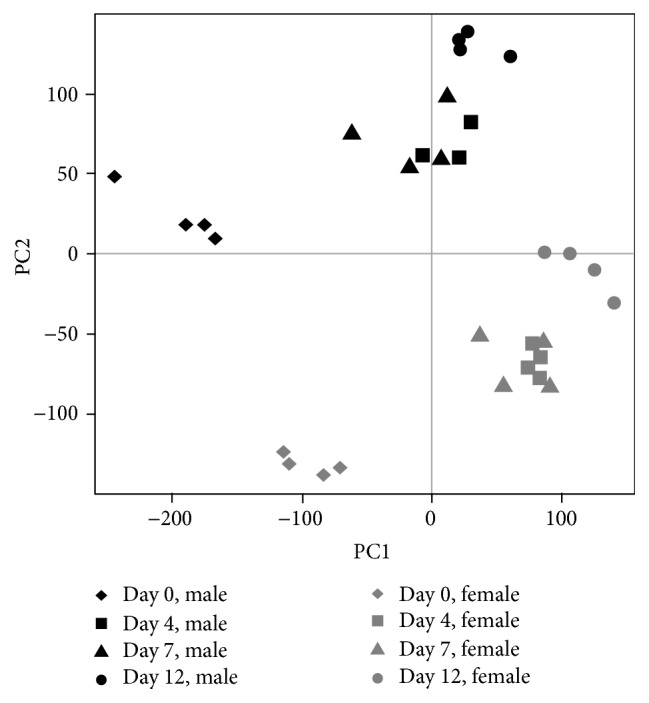
2D snapshot of PCA analysis of sample distributions based on all genes. The day 0 samples were well separated from the day 12 samples indicating dramatic transcriptomic changes during the dedifferentiation process. Moreover, the day 4 and day 7 samples were close together indicating small-magnitude transcriptomic changes between days 4 and 7.

The PCA was first performed to identify the variables driving gene expression variability through the dedifferentiation process (Figure 2) from normalized expression data of 33,297 transcripts with nonmissing data. Gene expression profiling at 4 time points allowed us to examine longitudinal variation in gene expression responses. Samples at day 0 were well separated from samples at day 12 suggesting important transcriptomic changes during the process. Day 4 and day 7 samples were close together, which could suggest small-magnitude transcriptomic changes between these two time points. Male and Female samples were separated even if they showed similar longitudinal changes during the dedifferentiation process. This difference was maintained even when we considered both fat depots separately.

### 3.3. Clustering Analysis

**Figure 3:**
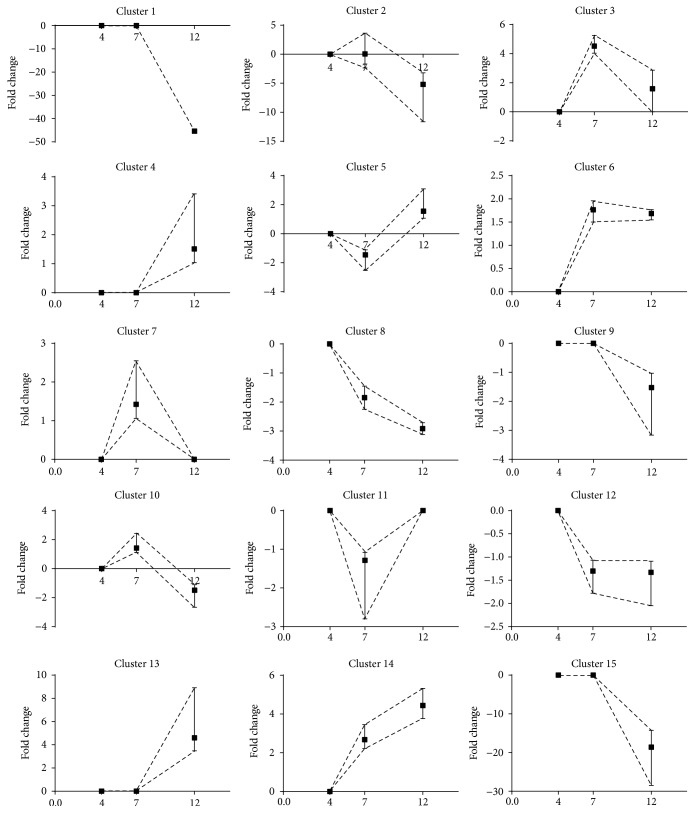
Cluster analysis according to changes in gene expression from day 4 to day 7 and from day 7 to day 12.

Hierarchical clustering was used to group differentially expressed transcripts with annotated genes in clusters according to similarity of changes in expression levels between dedifferentiation time points between day 4 and day 7 as well as between day 7 and day 12. We chose not to cluster genes according to changes in expression levels between day 4 and day 0 because we did not want to compare day 4 cells with uncultured mature adipocytes. We identified 15 different clusters that are presented in Figure 3. Clusters 1, 2, 9, and 15 included transcripts for which expression did not change from day 4 to day 7 but decreased from day 7 to day 12. The ADIPOQ transcript was the only one included in cluster one because its expression was drastically decreased from day 7 to day 12. Genes included in clusters 1, 2, 9, and 15 were related to mature adipocyte functions and lipid metabolism including triacylglycerol degradation and synthesis, lipolysis, lipid oxidation, and uptake of fatty acids (DGAT2, LIPE, PLIN1, PNPLA2, CEPB*α*, DGAT1, INSR, IRS2, NPY5R, IGF1, FABP4, LEP, and ADIPOQ). Clusters 3, 6, 7, and 10 included transcripts for which expression increased from day 4 to 7 and decreased from day 7 to day 12. Clusters 3 and 6 contained a few genes that were not related to specific cellular programs. Cluster 7 included genes related to cell cycle (CCNG1; ERCC1) and cellular growth (IGFBP4; IGFBP7). Genes related to the storage, breakdown, and secretion of lipids (CD36, LIPE, PLIN1, and FABP4) were included in cluster 10. In clusters 4 and 13, gene expression was stable from day 4 to day 7 but increased from day 7 to day 12. Genes included in cluster 4 and 13 were related to cell cycle and renewal of stem cells (CCNB1, CCNA2, FGF7, MAP2K4, WNT5A, KITGL, and FGF5). Clusters 5 and 11 grouped together transcripts for which expression decreased from day 4 to day 7 but increased from day 7 to day 12, whereas the expression of genes included in cluster 8 decreased from day 4 to day 7 and from day 7 to day 12. Genes included in cluster 5 were mostly related to cell cycle (CKAP2, ATG7, CENPE, CKAP2, ESCO2, RNF4, and XRCC2) while cluster 11 included genes related to stem cells and fibroblasts (CDC73, E2F3, RB1, and NFKBIA). Cluster 12 gathered together transcripts for which expression decreased from day 4 to day 7 but was stable from day 7 to day 12. Transcripts included in cluster 14 had increased expression from day 4 to 7 and from day 7 to day 12. Finally, genes included in cluster 12 showed a decrease in their expression from day 4 to day 7 but no change in their expression from day 7 to day 12. Genes included in this cluster were not related to a specific cellular program.

### 3.4. Enrichment Analysis

**Figure 4:**
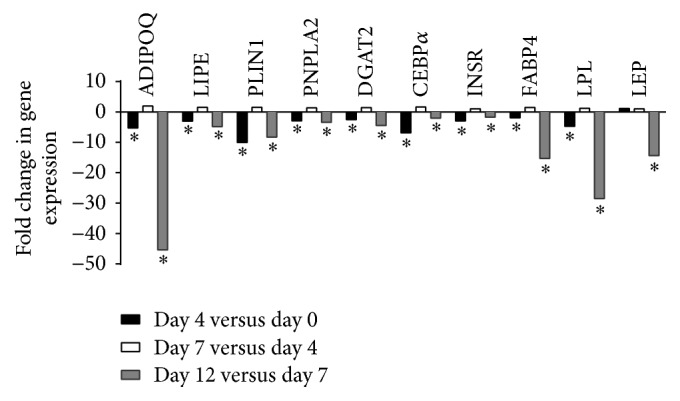
Fold changes in expression of genes related to adipogenic and lipogenic functions during the dedifferentiation process (^*∗*^*P* ≤ 0.05).

**Figure 5:**
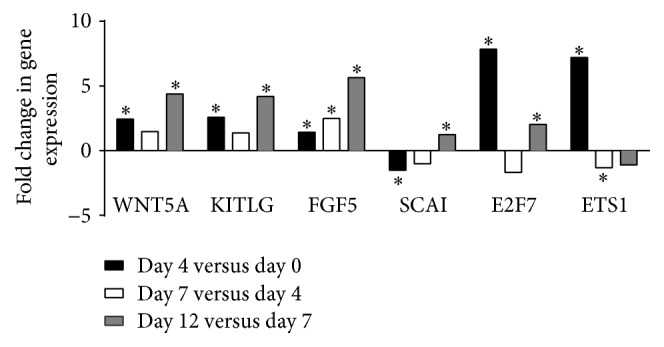
Fold changes in expression of genes related to cell cycle during the dedifferentiation process (^*∗*^*P* ≤ 0.05).

**Figure 6:**
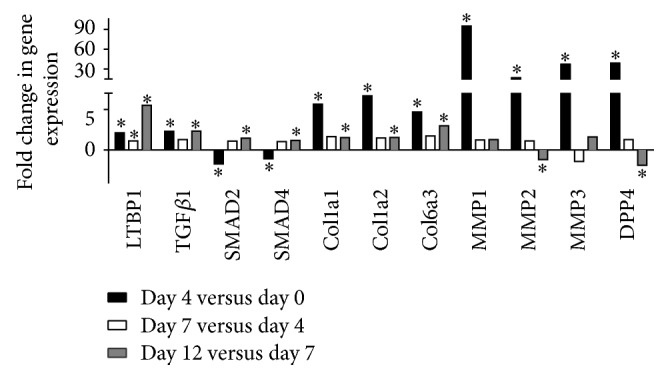
Fold changes in expression of genes related to extracellular matrix remodeling during the dedifferentiation process (^*∗*^*P* ≤ 0.05).

All clusters containing more than 10 genes were submitted to the Ingenuity Pathway system for gene function enrichment analysis. In cluster 2, the triacylglycerol degradation pathway was overrepresented (*P* = 4.48 × 10^−5^) while the lipid metabolism function was overrepresented and significantly decreased (*P* < 5.36 × 10^−11^–8.8 × 10^−3^). Consistently, in this cluster, gene expression of 52 genes coding for proteins involved in lipid metabolism and adipocytes functions (*LIPE*,* PLIN1*,* DGAT2*, and* PNPLA2*) was downregulated from day 7 to day 12. Clusters 1, 9, 10, and 15 also included genes coding for proteins related to the lipogenic and/or adipogenic functions (*ADIPOQ*,* CEBPA*,* LPL*,* FABP4*, stearoyl-CoA desaturase* (SCD)*, insulin receptor* (INSR)*, and* LEP*) of adipocytes. Fold changes in expression of these genes are shown in Figure 4.* ADIPOQ* showed the largest decrease in gene expression from day 7 to day 12 with a fold change of 40. Expression of this gene from day 0 (mature adipocytes) to day 4 was also significantly decreased but to a lesser extent.* LIPE*,* PLIN1*,* PNPLA2*,* DGAT2*,* CEBPA*,* INSR*,* FABP4*, and* LPL *gene expression were also significantly decreased from day 0 to day 4 and from day 7 to day 12.* LEP* gene expression significantly decreased when comparing day 12 to day 7. In clusters 4, 5, 7, 12, and 13, the molecular and cellular functions that were overrepresented were associated with cell cycle, cellular assembly and organization, cell morphology, cellular development, cell function and maintenance, and cell signaling. Fold changes in expression of genes coding for these functions are shown in Figure 5. The expression of* WNT5A*, Kit ligand* (KITGL), FGF5*, E2F transcription factor 7* (E2F7)*, and ETS protooncogene 1 transcription factor* (ETS1)* was significantly increased from day 4 compared to day 0 (mature adipocytes) and from day 12 compared to day 7. Gene expression of suppressor of cancer cell invasion* (SCAI)* was significantly decreased when comparing day 4 to day 0 and significantly increased when comparing day 12 to day 7. No significant change in gene expression was seen when comparing day 7 to day 4 except for the* FGF5* gene. We next examined fold changes in expression of transcripts related to the ECM during the dedifferentiation process.  Figure 6 shows a significant increase in gene expression of latent transforming growth factor beta binding protein 1* (LTBP1)*,* TGFβ1*,* SMAD2*,* SMAD4*,* COL1A1*,* COL1A2*, and* COL6A3* from day 12 compared to day 7 while a significant decrease in expression of* MMP2* and* DPP4* was observed for these time points. The expression of* LTPB1*,* TGFβ1*,* COL1A1*,* COL1A2*,* COL6A3*,* MMP1*,* MMP2*,* MMP3*, and* DPP4* significantly increased from day 0 to day 4. No significant change in gene expression was observed between day 4 and day 7 except for* LTBP1*.

### 3.5. In Vitro Validation Analysis

**Figure 7:**
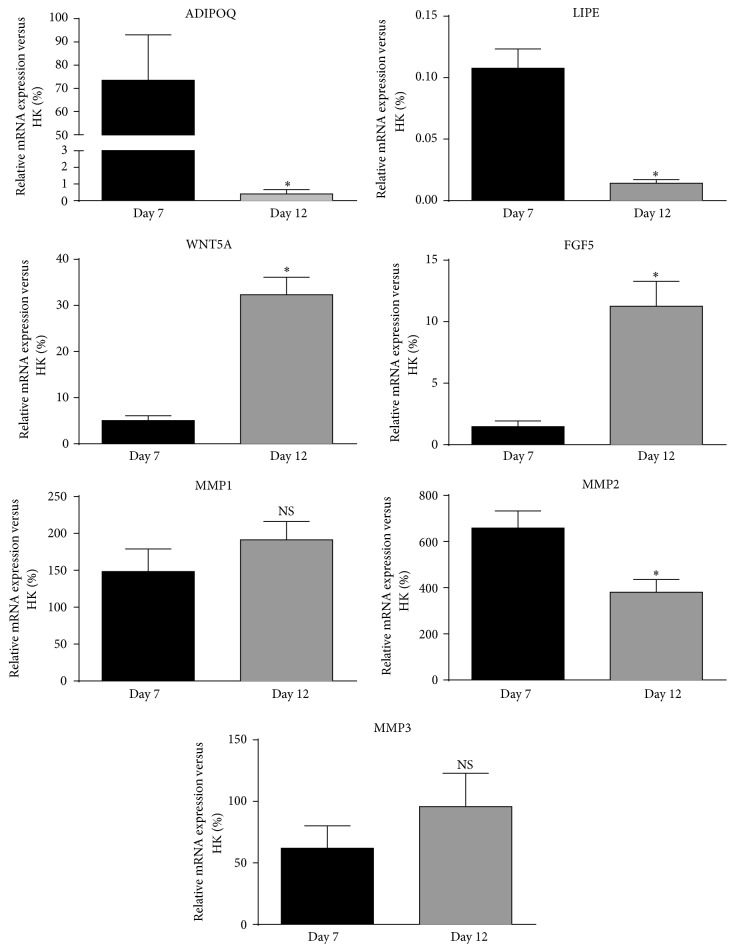
Differences in gene expression at day 7 and day 12 of the dedifferentiation process measured by quantitative RT-PCR. Target gene amplifications were normalized using housekeeping gene expression levels of ATP synthase O subunit (ATP5O). Values are mean ± SEM (^*∗*^*P* ≤ 0.05).

To confirm the results, we measured gene expression of transcripts that were modulated in a significant manner between day 7 and day 12 of the dedifferentiation process by quantitative RT-PCR. As shown in Figure 7, all transcripts were expressed at day 7 of the process.* ADIPOQ* and* LIPE* transcripts were strongly and significantly downregulated at day 12 compared to day 7 (*P* ≤ 0.05). A significant increase in gene expression of* WNT5A* and* FGF5 *was observed from day 7 to day 12 (*P* ≤ 0.05) but no significant change was observed for* MMP1*,* MMP2*, and* MMP3*, similar to observations in the microarray data.

## 4. Discussion

The aim of this study was to better understand human mature adipocyte dedifferentiation by characterizing changes in gene expression during this process. We obtained cells from morbidly obese male and female patients undergoing bariatric surgery and we successfully dedifferentiated fat cells from the OM and SC abdominal compartments. We then identified genes or groups of genes for which expression, assessed by Affymetrix microarrays, is modulated during dedifferentiation. We first demonstrated an important difference in transcriptomic profile of adipocytes (day 0) and dedifferentiated fat cells (day 12). A total of 15 different clusters were identified according to the similarity of changes in expression levels between dedifferentiation time points day 4 and day 7, as well as between day 7 and day 12. Functions that were overrepresented among these clusters were associated with downregulation of the adipogenic program and to upregulation in functions related to cell cycle, cell morphology, and cellular development. We also observed significant changes in the expression of genes related to the ECM during the dedifferentiation process.

We reported downregulation of transcripts encoding proteins involved in mature adipocyte functions. More specifically, we observed significant downregulation of the* ADIPOQ*,* LIPE*,* PLIN1*,* PNPLA2*,* DGAT2*,* CEBPA*,* INSR*, and* FABP4* transcripts from day 0 to day 4 and from day 7 to day 12. We have previously shown that, during the dedifferentiation process, the adipocyte cytoplasm flattens after 4 days of culture. We also demonstrated that lipid droplet size was statistically modulated by time during the process when assessed by red pixels after oil red O staining [2]. Other authors have previously shown that* PPARγ*,* CEBPA*,* LPL*, and* ADIPOQ* are downregulated once the cells are dedifferentiated [1, 5, 16]. Here we show that gene expression changes occur early in the process, suggesting that adipocytes can shut down their adipogenic program although many cells still have an adipocyte-like phenotype. This is consistent with our previous results showing a decrease in gene expression of* PPARγ2*,* CEBPA*,* LPL*, and* ADIPOQ* during the dedifferentiation process [2]. Here, we also observed important changes in gene expression of* ADIPOQ*,* LPL*, and* FABP4* at the end of the process, from day 7 to day 12.

Differentiation of preadipocytes into insulin-sensitive mature adipocytes producing adiponectin is stimulated by two families of transcription factors, the* CEBPs* and the* PPARs*. In vivo, it has been shown that* PPARγ* and* CEBPA* are critical regulators of adipogenesis [17–20] and that their presence is mandatory for the development of white adipose tissue [21]. In addition to promoting adipocyte differentiation,* PPARγ* and* CEBPA* allow sustaining and maintaining the mature adipocyte phenotype. We show that PPAR*γ* and CEBPA expression are downregulated during dedifferentiation, possibly because the commitment of mesenchymal stem cells to the adipogenic lineage requires these transcription factors. Similarly, adiponectin expression and secretion are specific to mature adipocytes. As a fat-derived hormone, adiponectin was shown to be an important messenger between adipose tissue and other organs [22]. The strong downregulation in adiponectin gene expression that we observed is concordant with loss of the mature adipocyte phenotype. Leptin is also mostly produced and secreted in adipocytes and its downregulation is also consistent with loss of the mature fat cell phenotype. Intravascular hydrolysis of triacylglycerol-rich lipoproteins is catalyzed by adipocyte-derived* LPL*, a process that has been tightly related to adipocyte size [23–25]. As* FABP4* plays a role in fatty acid uptake, downregulation in gene expression of this transcript is also consistent with loss of the mature adipocyte phenotype. Knockdown studies have shown that* PPARγ*,* CEBPA*, and* CEBPB* are all required to sustain the expression of their target genes in mature adipocytes including adiponectin,* FABP4*, and hormone-sensitive lipase. This could explain the concomitant decrease in gene expression of* FABP4* as the dedifferentiation process takes place [26].

In addition to the morphological changes seen during dedifferentiation, we also observed a release of oil in the culture media, suggesting that adipocytes shed their lipids (not shown). Still, little is known about the physiological process of dedifferentiation and how the cells lose their lipid content. We observed a significant decrease in gene expression of proteins implicated in lipolysis. Regulation of lipolysis occurs at multiple levels and involves many proteins. Among these are the aquaglyceroporins, a family of glycerol-transporting proteins expressed in the plasma membrane of adipocytes. These proteins are channels for the transport of glycerol across the membrane of adipocytes [27]. We observed a significant decrease in gene expression of AQP7 from day 0 to day 4 and from day 7 to day 12. This is concordant with the downregulation of the adipogenic program and with the observed changes in the size of the lipid droplets. As lipolysis proceeds in a regulated manner, it would be interesting to examine changes in expression of other molecules implicated in this process. It has recently been suggested that isolated mature adipocytes transdifferentiated spontaneously to fibroblast-like cells in vitro, a process that involves liposecretion [28]. More studies are needed to understand the process of dedifferentiation and to examine if it involves lipolysis or liposecretion.

We observed an increase in the expression of genes coding for proteins implicated in processes such as cellular development, cell cycle, cell development, and cell signaling. We report an increase in gene expression of* WNT5A* and* E2F7* in both the early and the late stages of dedifferentiation.* E2F7* plays an essential role in the regulation of cell cycle progression [29].* ETS1* is a transcription factor that regulates numerous genes and is involved in stem cell development while* WNT5A* is part of a large secreted protein family that controls essential developmental processes such as embryonic patterning, cell growth, migration, and differentiation. Recently, Zamboni et al. studied the crosstalk between adipocytes and pancreatic cancer cells and its consequences on the tumor microenvironment. Their data suggested the existence of a process characterized by adipocyte dedifferentiation/reprogramming toward fibroblast-like cells that would be mediated by* WTN5A* [30]. In line with our results, several authors have demonstrated that dedifferentiated mature adipocytes have the molecular signature of a reprogrammed cell showing features similar to stem cells [5, 16, 31]. Because genes associated with the adipogenic program are downregulated and genes involved in cell proliferation are upregulated, our results suggest that mature adipocytes may have reentered the cell cycle and that gene-reprogramming events are taking place. Zhang et al. demonstrated that cultured mature adipocytes incorporate 3H-thymidine and BrdU into their nuclei, indicating that adipocytes enter the S phase of the cell cycle during ceiling culture [6]. Nobusue et al. also demonstrated that mature adipocytes enter the cell cycle, dedifferentiate into fibroblast-like cells, and proliferate in ceiling culture [32].

We have previously shown that genes associated with the ECM were strongly upregulated in dedifferentiating adipocytes. Specifically, we demonstrated that gene expression of* MMP1*,* FAP*,* DPP4*, and* TGFβ1* were strongly induced during dedifferentiation [2]. Our results corroborate these findings as we observed an increase in gene expression of* LTPB1*,* TGFβ1*,* SMAD2*,* SMAD4*,* COL1A1*,* COL1A2*,* COL6A3*,* MMP1*,* MMP2*,* MMP3*, and* MMP4*. Gene expression of all three collagen isoforms and the 4 endopeptidases MMPs increased early and extensively during the process.* TGFβ* is known to be a potent inducer of ECM protein-coding genes such as the collagens [33]. The TGF*β*-mediated increase in collagen gene expression has been demonstrated in several studies [34, 35]. The TGF*β* pathway sends signals via phosphorylation of the receptor-regulated SMADs (R-SMADs), which can bind the* coSMAD*,* SMAD4* [36, 37]. The increase in gene expression of* SMAD2* and* SMAD4* we observed is in line with an activation of the TGF*β* pathway. However, because we did not measure the protein and phosphorylation levels of the receptor SMADs, more studies are needed to confirm activation of this pathway. Adipose tissue continuously undergoes a process of remodeling referring to the notion of adipocyte plasticity. This includes turnover of adipocytes and reorganization of the ECM in response to changes in the environment of the tissue [38, 39]. Consistent with our previous study,* MMP1* gene expression was upregulated during the dedifferentiation process. Perrini et al. observed higher expression of* MMP3* in DFAT cells compared to adipose-derived stem cells [31]. Furthermore, others have shown that some MMPs and their inhibitors (TIMPs) are modulated with obesity level in mice and in humans and that* MMP2* and* MMP9* are essential for adipocyte differentiation [40, 41]. These results suggest that other MMPs could be involved in reversing the differentiation process. The changes in gene expression of these enzymes during dedifferentiation in addition to the morphological changes we observed also support a role for the MMPs in this process. We postulate that a remodeling of the ECM is mandatory for adipocytes to dedifferentiate.

Taken together these results demonstrate important modifications in the cellular program of mature adipocytes undergoing dedifferentiation in the early stage of the process, as well as later in the culture. We observed a downregulation of mature adipocyte transcripts in addition to strong upregulation in the expression of transcripts coding for matrix remodeling proteins and an increase in transcripts coding for proteins of cellular development, cell cycle, cell morphology, and cell signaling. To the best of our knowledge, we are the first to investigate transcriptomic changes in OM and SC human mature adipocytes undergoing dedifferentiation. We studied the process longitudinally instead of focusing exclusively on the resulting DFAT cells. The current study has some limitations that need to be acknowledged. These analyses were performed in morbidly obese individuals. Hence, we cannot extrapolate the results to individuals with less pronounced obesity levels or to the general population. Differences in gene expression of lipogenic enzymes have been shown previously. Ortega et al. demonstrated that expression of lipogenic enzymes is downregulated in visceral adipose tissue of obese subjects [42]. The expression of genes involved in energy homeostasis and those encoding growth factors is also regulated in a different manner between obese and lean people. Genes related to lipolysis are upregulated in obesity while genes encoding growth factors are downregulated [43]. Concordant with this finding, it is possible that the level of expression we observed for some enzymes is different from the one we would observe in less obese people. Still, we think that the pattern of changes we observed would be similar. In addition, we have successfully dedifferentiated cells from men and women covering a wide range of adiposity and age, as well as individuals with or without type 2 diabetes. The design of this study has a validity that needs to be acknowledged. Hybridization of cRNA on Affymetrix was performed for each individual, in each depot at each time point during the dedifferentiation process for a total of 64 arrays. Plus, each probe interrogated >36,000 transcripts and targeted >21,000 RefSeq genes. Finally, our results were confirmed with RT-PCR.

## Supplementary Material

Adipose tissue samples were obtained from 2 men and 2 women undergoing bariatric surgery as a treatment for severe obesity. They were paired for age and BMI (mean age: 51 years; mean BMI: 48 kg/m^2^).
