# Supplementary material for: Temporal Changes in Gene Expression Profile during Mature Adipocyte Dedifferentiation
Source: Int J Genomics. 2017 Mar 19;2017:5149362. doi: 10.1155/2017/5149362 (PMC5376413; doi:10.1155/2017/5149362)
Supplement: Supplementary file 1 — Adipose tissue samples were obtained from 2 men and 2 women undergoing bariatric surgery as a treatment for severe obesity. They were paired for age and BMI (mean age: 51 years; mean BMI: 48 kg/m2). [file 5149362.f1.pdf]

Supplemental Table 1. Blood lipid profile of the patients involved in the study

Patients	Total cholesterol (mmol/L)	Triglycerides (mmol/L)	HDL-cholesterol (mmol/L)	LDL-cholesterol (mmol/L)	Total cholesterol/HDL-cholesterol	HbA1c (%)
1	3.38	2.22	0.7	1.67	2.02	6.5
2	3.39	1.86	1.13	1.42	3.00	5.9
3	5.57	1.49	1.48	3.41	3.76	6.2
4	5.14	1.21	1.1	3.39	4.67	6.4

*Patients 1 and 3 were taking medication for dyslipidemia. Patients 1.2 and 3 were taking medication for hypertension and were suffering from sleep apnea. All patients were non-smokers. Patients 1,2 and 4 were diabetic. Patient 1 was taking oral medication. patient 2 was treated with insulin and patient 4 did not take any medication.
